# Effect of domestic COVID-19 vaccine on the plasma D-dimer levels of early pregnant women in China

**DOI:** 10.3389/fmed.2023.1219502

**Published:** 2023-09-01

**Authors:** Wenjuan Liang, Xin Fu, Rui Li, Liu Yang, Peng Liu, Xuan Guo, Qinliang Jia, Ziran Wang, Yun Xie

**Affiliations:** ^1^Medical Laboratory Center, Northwest Women’s and Children’s Hospital, Xi'an, China; ^2^Department of Laboratory Medicine, Peking Union Medical College Hospital, Chinese Academy of Medical Sciences, Beijing, China

**Keywords:** D-dimer, COVID-19 vaccine, early pregnant women, domestic vaccine, China

## Abstract

**Objective:**

To investigate the effect of COVID-19 vaccination on plasma D-dimer levels in early pregnant women.

**Methods:**

A total of 834 early pregnant women(gestational age ≤ 13 weeks), who visited Northwest Women and Children’s Hospital between December 2020 and April 2022, were selected. There were 696 women in the healthy group (group A) and 138 in the group with a history of adverse pregnancy and childbirth (group B). The plasma D-dimer levels of all participants were tested, and the COVID-19 vaccine history of all participants was collected using a survey questionnaire.

**Results:**

The plasma D-dimer levels did not differ between group A and the group B (*p* = 0.1327). In the group A, 470 were vaccinated and 226 were unvaccinated. The D-dimer levels of vaccinated individuals were lower than those of unvaccinated individuals (*p* = 0.0047). In the group B, 84 were vaccinated and 54 were unvaccinated; no difference in D-dimer levels was found between the vaccinated and unvaccinated individuals (*p* = 0.0542). In the group A, the D-dimer levels of the unvaccinated group were not different from those of women vaccinated with one dose (*p* = 0.208), but they were higher than those who received two doses (*p* = 0.019) or three doses (*p* = 0.003). And, no significant difference in D-dimer levels was found among women who received different vaccine brands and with different vaccination times.

**Conclusion:**

This study preliminarily indicates that COVID-19 vaccination does not increase D-dimer levels in early pregnant women.

## Introduction

Since the end of December 2019, the coronavirus disease 2019 (COVID-19) pandemic caused by severe acute respiratory syndrome coronavirus 2 (SARS-CoV-2) has spread worldwide. According to the World Health Organization (WHO) website, as of February 19, 2023, more than 757 million confirmed cases and more than 6.8 million deaths have been reported worldwide. Vaccination with a COVID-19 vaccine is currently an effective means to control COVID-19 because it can effectively reduce the SARS-CoV-2 infectivity rate and the COVID-19 fatality rate (1). At present, the COVID-19 vaccines that have been successfully developed and marketed include inactivated vaccines (inactivated SARS-CoV-2), virus-like particle vaccines (virus particles without nucleic acid), subunit vaccines (*in vitro*-expressed spike (S) protein or receptor binding domain (RBD)), viral vector vaccine (replication-deficient engineered virus carrying S protein or RBD mRNA), DNA vaccines (S protein or RBD DNA sequence), mRNA vaccines (S protein or RBD RNA sequence), and live attenuated vaccines. These vaccines induce the production of neutralizing antibodies that protect the recipient from viral infection (2). The COVID-19 vaccines have shown good efficacy and safety in clinical trials and real-world studies (3). According to the WHO, as of February 22, 2023, more than 1.32 billion doses of COVID-19 vaccines have been administered worldwide. The main domestic vaccines in China that have been marketed include CoronaVac (Sinovac), BBIBP-CorV, ZF2001 (Anhui Zhifei Longcom), and Convidecia (Cansino). Among them, CoronaVac and BBIBP-CorV are inactivated vaccines in which the virus has been inactivated by physical or chemical methods, but the immunogenicity of the virus is retained and can elicit an immune response. ZF2001 is a recombinant protein vaccine based on protein subunits; it contains residues 319–545 of the SARS-CoV-2 spike protein RBD. After vaccination, an immune response is produced that blocks binding between the RBD and ACE2, which is expressed on the cell surface. This vaccine was shown to neutralize infection by SARS-CoV-2 pseudovirus and live virus *in vitro*. Convidecia is a recombinant type 5 adenovirus vector vaccine. It uses a human replication-deficient adenovirus vector in which the genes related to adenovirus replication have been removed and the SARS-CoV-2 S protein gene has been inserted. After vaccination with this recombinant virus vaccine, the immune system is stimulated and mounts an immune response, generates memory, and quickly responds to clear SARS-CoV-2. According to the Chinese Center for Disease Control and Prevention, as of February 23, 2023, a total of 3.492329 billion doses of COVID-19 vaccines have been administered in China. In total, 1.310406 billion people have been vaccinated. The first dose and full course vaccination coverage rates of the whole population are 93.0 and 90.6%, respectively.

Despite these positive results, international studies have reported on the rare occurrence of vaccine-induced immune thrombocytopenia and cerebral venous sinus thrombosis after vaccination with the COVID-19 vaccines (4, 5). These complications are very rare but are serious and potentially fatal (5). Currently, no clear evidence proves that vaccination leads to an increased risk of thrombotic events. However, data related to the long-term safety of the vaccines, their interactions with other vaccines, and their use in pregnant or lactating women, immunocompromised patients, and other vulnerable subgroups, are missing (6). Thus, the use of these vaccines in women of reproductive age and pregnant women, immunocompromised patients, and other vulnerable subgroups needs to be tracked and studied. The benefits and risks of COVID-19 vaccines must be weighed against the likelihood of infection, the development of complications, and long-term sequelae.

Previous studies have found an increased risk of SARS-CoV-2 infection and admission to the ICU at any point during pregnancy, and the overall ICU transfer rate is approximately 3%. SARS-CoV-2 infection carries a significant risk of maternal mortality. Complications of COVID-19 include preeclampsia, preeclampsia-eclampsia, and thrombotic disorders. Compared with non-SARS-CoV-2-infected babies, babies with the infection have an increased risk of transfer to the neonatal ICU. Pregnant women with SARS-CoV-2 infection have a three-fold increased risk of moderate preterm birth (i.e., births occurring at 32–34 weeks), and a two-fold increased risk of preterm birth (7) compared with non-SARS-CoV-2-infected pregnant women. Therefore, for pregnant women or those preparing for pregnancy, trying to prevent SARS-CoV-2 infection to reduce the risk of adverse pregnancy outcomes is important. For this population, being vaccinated with a COVID-19 vaccine can be an effective measure. However, further studies should be conducted on the safety and efficacy of vaccines in pregnant women and those preparing for pregnancy.

D-dimer is a specific fibrin degradation product that is produced by plasmin hydrolyzing the cross-linked fibrin monomers. An increased D-dimer level indicates hypercoagulability and secondary hyperfibrinolysis (8). Abnormally increased D-dimer levels in pregnant women suggest an increased risk of disseminated intravascular coagulation and indicate an increased probability of adverse pregnancy outcomes (9). A negative D-dimer test during pregnancy can safely rule out suspected deep vein thrombosis (10). The D-dimer level in the second trimester has a high predictive value for deep vein thrombosis (11). Therefore, monitoring the plasma D-dimer level in pregnant women is helpful for assessing the risk of thrombotic diseases during pregnancy. This study was conducted to investigate the effect of COVID-19 vaccination on the plasma D-dimer levels of pregnant women in the first trimester. The findings are intended to guide the rational use of vaccines.

## Materials and methods

### Research participants

A total of 834 early pregnant women who visited Northwest Women and Children’s Hospital between December 2020 and April 2022 were selected. We obtained information about their vaccination history through survey questionnaires and telephone surveys. The inclusion criteria were as follows: early pregnant women over the age of 18 years who agreed to participate; early pregnancy was defined as a gestational age ≤ 13 weeks based on the literature (12). The exclusion criteria were as follows: women who were taking any drug that may affect the fibrinolytic system or coagulation function, or having a personal or family history of coagulation, malignant tumors, or autoimmune disease. The group of healthy pregnant women included 696 individuals (group A), aged 20–44 years, with a median age of 30 years. This group had no history of adverse pregnancies and had regular prenatal examinations during pregnancy with normal results. The group with a history of adverse pregnancy and childbirth included 138 pregnant women (group B), aged 22–42 years, with a median age of 31 years. The adverse outcomes mainly included a history of spontaneous abortion, missed abortion, premature delivery, fetal termination, stillbirth, birth of a child with a serious birth defect, neonatal death within 28 days of birth, and diabetes or hypertension during pregnancy. The vaccination information collected in the survey questionnaire included the vaccine brand, timing of vaccination, and number of doses.

### Instruments and reagents

The fully automatic coagulation instrument STA-R Evolution (Diagnostica Stago S.A.S., Asnières sur Seine, France) was used with its original matching reagents, calibrators, and quality control products.

### Sample collection and testing

In the morning, 1.8 mL of venous blood was drawn from each participant on an empty stomach and put into a 109 mmol/L sodium citrate anticoagulation tube and centrifuged at 2,360 *× g* for 10 min to obtain platelet-depleted plasma. There was no hemolysis, jaundice, or chyle in any of the plasma samples, and the D-dimer testing was completed within 2 h. Quality control samples with high and low concentrations were prepared every day, and the samples were tested after the quality control parameters had been met.

### Statistical analysis

Statistical analysis and plotting were conducted using SPSS 20.0 and GraphPad Prism 9.0 software. The quantitative data were first subjected to normality testing. The Kolmogorov–Smirnov test was used to test the normality of “age” and “D-dimer.” Data with a skewed distribution are represented by the median (quartile interval) [M (P_25_, P_75_)]. Two groups were compared using the Mann–Whitney test, and multiple groups were compared using the Kruskal–Wallis test. The qualitative data are represented by the number (percentage) [*n* (%)], and qualitative data of two independent samples were compared using the *χ*^2^ test. *p* < 0.05 was considered to indicate a statistically significant difference.

## Results

### Vaccination rate of pregnant women

Among the 834 pregnant women included in the study, the vaccination rate was 66.43% (554/834). The vaccination rate in the group A was 67.52% (470/696), and the vaccination rate in the group B was 60.87% (84/138). Chi-square test (*χ*^2^) was performed to evaluate the vaccination rate between group A and group B, the result showed there is no significant difference (*χ*^2^ = 2.290, *p* = 0.130).

### Comparison of the percentage of women that had normal D-dimer levels for each of the two groups

At present, the reference range of D-dimer commonly used in clinical practice is the average level of healthy adults. In this study, the diagnostic definition of D-dimer recommended by the Kit manual was 0.5 μg/mL (cut-off value: ≤0.5 μg/mL). The percentage of women that had normal D-dimer levels for each of the two groups was calculated by *χ*2 test, the result showed there is no significant difference between group A and Group B (*χ*2 = 0.105, *p* = 0.746).

### Comparison of clinical characteristics and D-dimer levels

As shown in Table 1, the average age in the group of healthy pregnant women was 30 (28, 32) years, which was lower than 31 (29, 34) years for the group with a history of adverse pregnancy (*p* = 0.0001). There was a significant difference in the number of pregnancies and birth frequency (*p* < 0.05) between the two groups, but there was no significant difference in gestational age.

**Table 1:** Comparison of clinical characteristics between the group A and the group B.

Clinical characteristics	Group A (*n* = 696)	Group B (*n* = 138)	*p* value
Age [year, M (Q1,Q3)]	30 (28, 32)	31 (29, 34)	**0.0001**
Number of pregnancies [times, *n* (%)]			**<0.001**
0	112 (16)	8 (6)	
1	348 (50)	42 (31)	
2	185 (27)	57(42)	
3	29 (4)	23 (17)	
4	12 (2)	6 (4)	
5	7 (1)	1(0)	
6	1 (0)	1(0)	
7	2 (0)	0(0)	
Birth frequency [times, *n* (%)]			**0.018**
0	483 (69)	78 (57)	
1	198 (29)	56(41)	
2	14 (2)	4 (2)	
3	1 (0)	0 (0)	
Gestational age [week, *n* (%)]			**0.887**
≤7	10 (1)	1(1)	
7^+1^ ~ 10^+6^	27 (4)	6(4)	
11 ~ 13^+6^	659 (95)	131(94)	
D-dimer level [μg/ml, M (Q1,Q3)]			
	0.32 (0.25, 0.44)	0.30 (0.23, 0.45)	**0.1327**

Group A: Group of healthy pregnant women. Group B: Group with a history of adverse pregnancy and childbirth. Bold values represent the *p* value of the Mann–Whitney test or Chi-square test between group A and B.

The average D-dimer level in the group of healthy pregnant women was 0.32 (0.25, 0.44) μg/mL, whereas it was 0.30 (0.23, 0.45) μg/mL in the group with a history of adverse pregnancy, but the difference was not significant (*p* = 0.1327).

### Effect of COVID-19 vaccine on D-dimer levels

**Figure 1:**
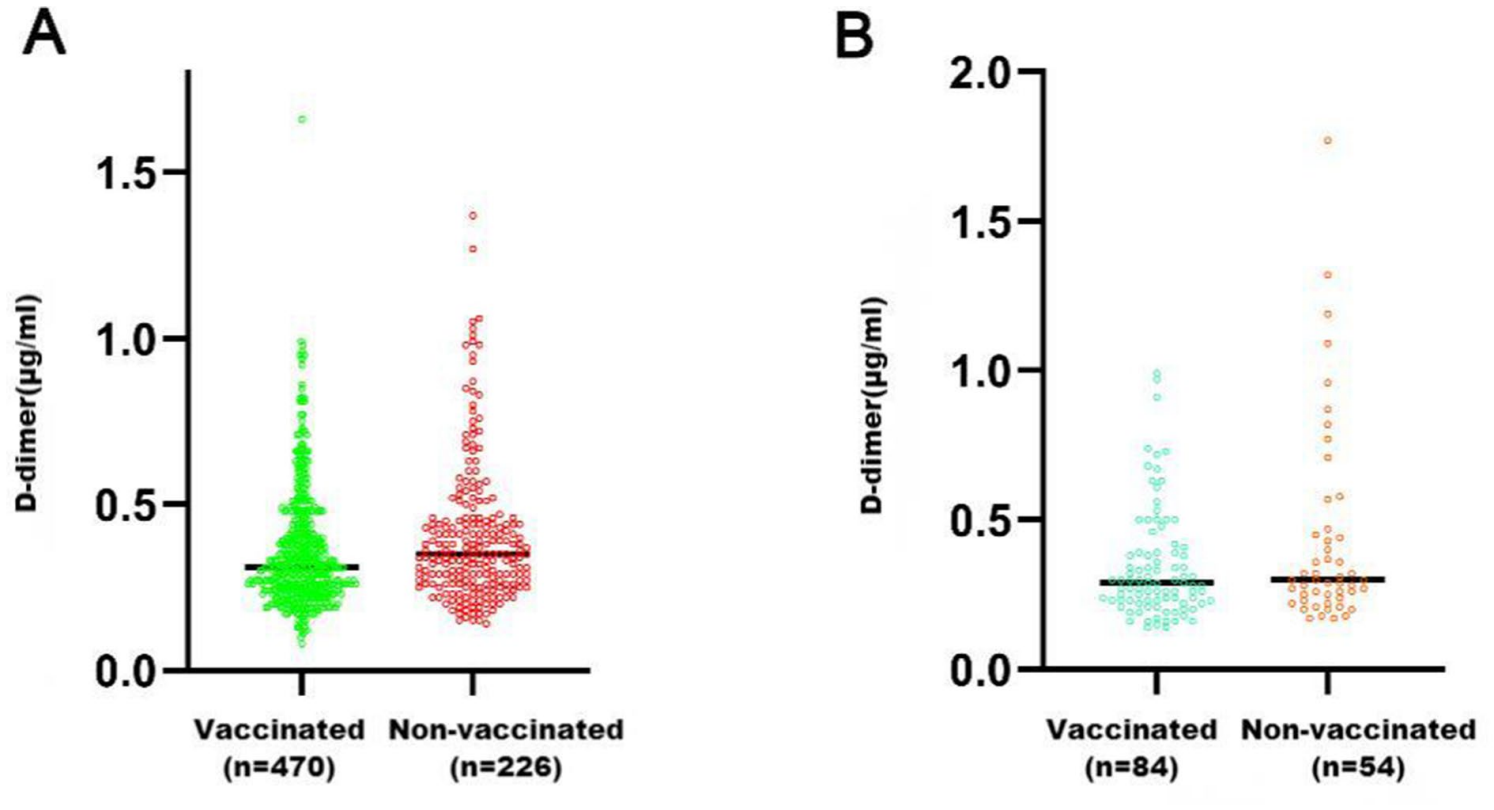
Comparison of D-dimer levels between pregnant women who received COVID-19 vaccination and those who did not. **(A)** Group of healthy pregnant women (Group A); **(B)** Group with a history of adverse pregnancy and childbirth (Group B). ^**^p < 0.01; ^*^p < 0.05; ns, *p* > 0.05.

The group of healthy pregnant women included 696 people; 470 were vaccinated and 226 were unvaccinated. The average D-dimer level in the vaccinated women [0.31 (0.25, 0.42) μg/mL] was lower than that in the unvaccinated women [0.35 (0.26, 0.46) μg/mL], and the difference was significant (*U* = 46,207, *p* = 0.0047) (Figure 1A). The group with a history of adverse pregnancy and childbirth included 138 pregnant women, with 84 vaccinated and 54 unvaccinated. The average D-dimer level of the vaccinated women was 0.28 (0.23, 0.41) μg/mL, and that in the unvaccinated women was 0.32 (0.26, 0.48) μg/mL; the difference was not significant (*U* = 1,827, *p* = 0.0542) (Figure 1B).

### Effect of COVID-19 vaccine dose on D-dimer levels in healthy pregnant women

**Figure 2:**
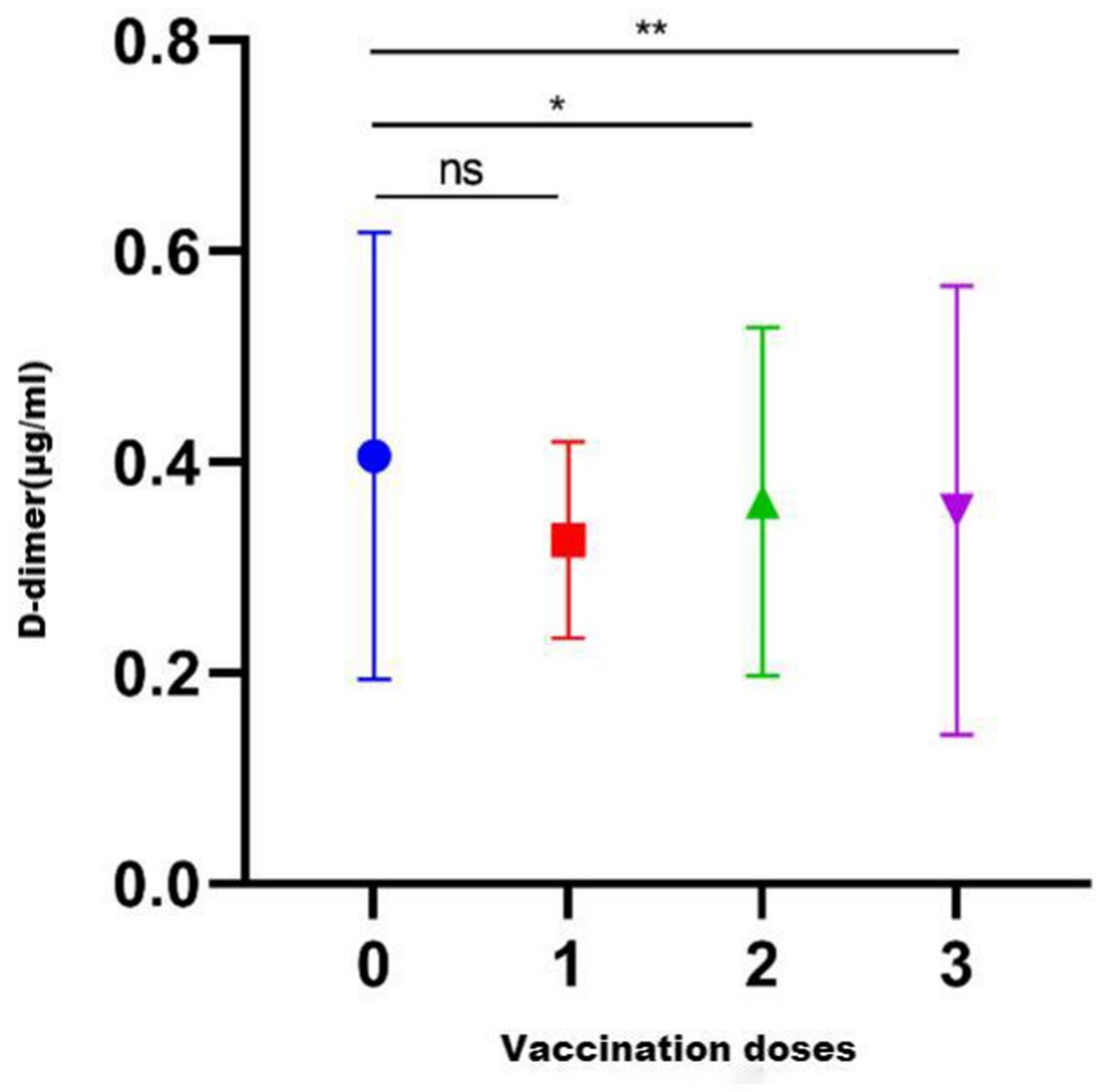
D-dimer levels after vaccination with different COVID-19 vaccine brand combinations. ns, *p* > 0.05.

Overall, 226 healthy pregnant women had never received a COVID-19 vaccination, whereas 470 had been vaccinated with a COVID-19 vaccine. Among the vaccinated women, 14 had received one dose, 347 had received two doses, and 109 had received three doses. There was significant difference in D-dimer levels among the different dose groups (*p* = 0.013). The D-dimer levels of the unvaccinated group were not different from those of women vaccinated with one dose (*p* = 0.208), but they were higher than those who received two doses (*p* = 0.019) or three doses (*p* = 0.003) (Figure 2).

### Effect of COVID-19 vaccine brand on D-dimer levels in healthy pregnant women

**Figure 3:**
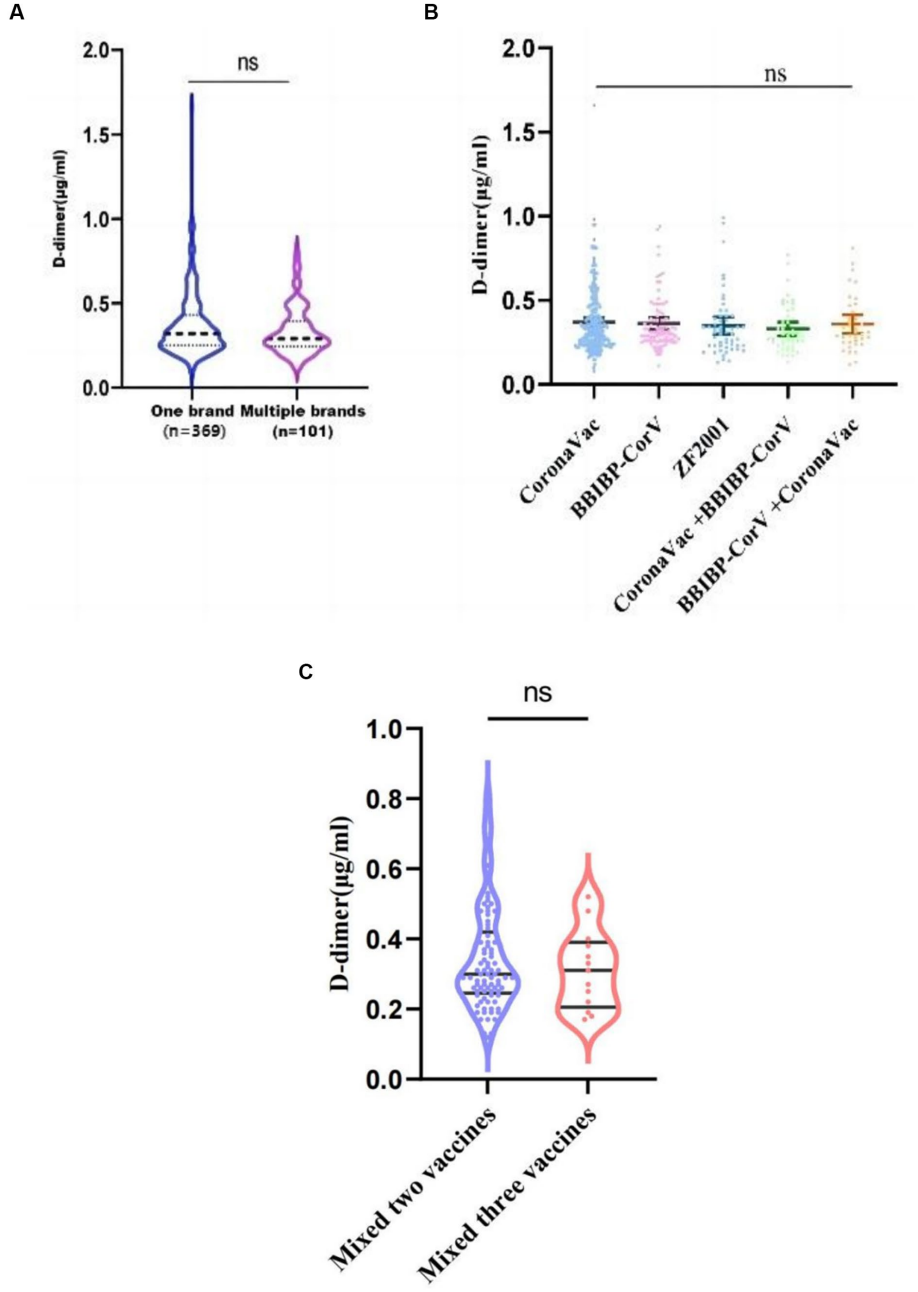
D-dimer levels among COVID-19 vaccine dose groups. **(A)** Average D-dimer levels for those who received a single vaccine brand vs. mixed brands; **(B)** Average D-dimer levels among those who received one of the five most common vaccine combinations. **(C)** Average D-dimer levels for those who vaccinated with two brands vs. vaccinated with three brands, ns, *p* > 0.05.

The brands of COVID-19 vaccines administered to the 470 healthy pregnant women are shown in Table 2. Among them, 369 women received the same brand for all doses, and this group had an average D-dimer level of 0.32 (0.25, 0.43) μg/mL; 101 women received different vaccine brands throughout the vaccination series, and this group had an average D-dimer level of 0.29 (0.25, 0.40) μg/mL. There was no significant difference between these two groups (*p* = 0.2268) (Figure 3A). There were also no significant differences in D-dimer levels among pregnant women who received one of the five most common vaccine combinations (*p* = 0.5976) (Figure 3B). As can be seen from Table 2, the number of people who received a single brand of vaccine was 369, and the number of people who received a combination of different brands of vaccine was 101 (including two brands and three brands). Taking into account the statistical impact of the sample size, a comparison was made between people vaccinated with two brands and those vaccinated with three brands. As shown in Figure 3C, there was no significant difference between the two groups (*p* = 0.6577).

**Table 2:** COVID-19 vaccine brands administered to healthy pregnant women.

Vaccine combination	Number of vaccinations
CoronaVac	231
BBIBP-CorV	81
ZF2001	54
CoronaVac +BBIBP-CorV	48
BBIBP-CorV +CoronaVac	37
BBIBP-CorV +CoronaVac +CoronaVac	5
CoronaVac +BBIBP-CorV+BBIBP-CorV	4
CoronaVac +CoronaVac +BBIBP-CorV	4
BBIBP-CorV +CoronaVac +BBIBP-CorV	3
Convidecia	3

The above grouping takes into account the order of vaccination for different brands.

### Effect of vaccination timing on D-dimer levels in healthy pregnant women

**Figure 4:**
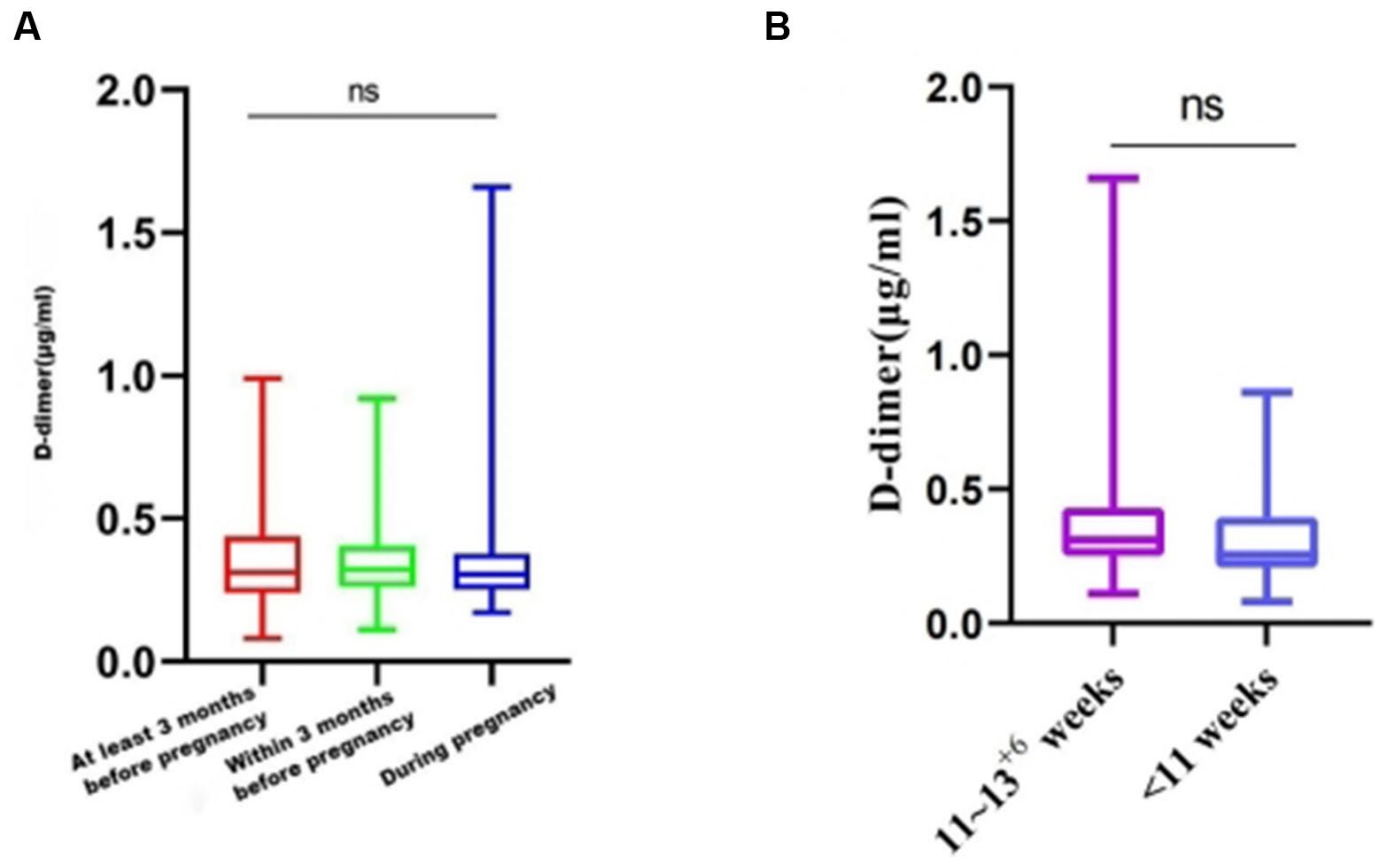
D-dimer levels among women with different COVID-19 vaccination times. **(A)** Average D-dimer levels for those different vaccination times. **(B)** Average D-dimer levels of vaccination at different gestational weeks. ns, *p* > 0.05.

The healthy pregnant women who were vaccinated were grouped according to the vaccination timing: 375 women were vaccinated at least 3 months before pregnancy, 53 women were vaccinated within 3 months of pregnancy, and 42 women were vaccinated during pregnancy. No significant difference in D-dimer level was found between the groups with different vaccination times (*p* = 0.9202) (Figure 4A).According to the gestational age, the vaccinated pregnant women in the first trimester were divided into 11–13^+6^ weeks and < 11 weeks, and the D-dimer values of the two groups were compared. The results showed no significant difference between the two groups (*p* = 0.0905) (Figure 4B).

## Discussion

COVID-19 vaccination is an important measure to prevent SARS-CoV-2 infection. Vaccination with an effective and safe vaccine plays a vital role in reducing the rate of severe cases and the mortality rate from SARS-CoV-2 infection and its complications. Herd immunity can be realized, which will help to quickly restore normal life and the normal operation of the global economy and will minimize the loss of people’s physical, social, and mental health caused by the global COVID-19 pandemic. Lin Y et al. assessed the public’s willingness to be vaccinated and the willingness to pay for vaccination to better understand the demand and hesitation related to the COVID-19 vaccines. The authors also investigated the public’s confidence in the COVID-19 vaccines produced in China and the preference for domestic or foreign vaccines. On the basis of 3,541 completed surveys, 54.6% of respondents were willing to potentially be vaccinated, while only 28.7% explicitly agreed to vaccination. A total of 48.7% of respondents expressed confidence in the domestic vaccines, 46.1% expressed that they had full confidence, and 64.2% expressed their preference for a domestic vaccine (13). In a national survey conducted in the United States by Kaplan RM et al. in 2020, only approximately one-third of Americans said they would likely receive a COVID-19 vaccine, and approximately one-fifth of adults said they likely would not receive vaccination under any circumstance. People’s hesitancy about vaccination might arise from the following concerns: doubts about whether the vaccine can effectively protect the body from SARS-CoV-2 infection or reduce the risk of severe illness; vaccination possibly inducing physical discomfort (e.g., fever, arm pain); and vaccination possibly causing other diseases or serious adverse reactions (such as temporary or permanent paralysis) (14). In recent years, adverse events caused by vaccination against the novel coronavirus have occurred frequently, with reported patients including children, the older population, and those undergoing assisted reproduction. Hause AM et al. reported that approximately 8.7 million doses of the Pfizer-BioNTech COVID-19 vaccines were administered to children aged 5–11 years in the United States between November 3 and December 19, 2021. The Vaccine Adverse Event Reporting System received 4,249 reports of adverse events following the Pfizer-bioNTech COVID-19 vaccination rollout, of which 4,149 (97.6%) were not serious adverse events. Approximately 42,504 children aged 5–11 years were included in V-Safe after receiving the Pfizer-bioNTech vaccine; a total of 17,180 (57.5%) local reactions and 12,223 (40.9%) systemic reactions (including injection site pain, fatigue, and headache) were reported after the second dose (15). The team led by Shi Juanzi explored the time interval between the first administration of an inactivated COVID-19 vaccine and *in vitro* fertilization (IVF) treatment and the pregnancy rate after fresh embryo transfer. A total of 3,052 female patients aged 20–47 years who underwent IVF during 2021 and were followed up until March 31, 2022, were analyzed. The results showed that the administration of the first dose of inactivated COVID-19 vaccine 60 days or less before fertilization therapy was associated with a decreased pregnancy rate. For IVF patients who will receive fresh embryo transfers, the procedure may need to be postponed until at least 61 days after COVID-19 vaccination because vaccination may reduce the pregnancy rate of assisted reproductive technology (16). Waheed et al. reported a case study of a 57-year-old woman who developed neuropathy (small fiber neuropathy) 1 week after receiving a second dose of Pfizer’s COVID-19 vaccine. Her neuropathic pain disappeared after treatment (17). Eid et al. reported a 79-year-old male patient who experienced varicella-zoster virus reactivation following vaccination with an mRNA COVID-19 vaccine. The patient had a history of hypertension, coronary artery disease, and antineutrophil cytoplasmic antibody-associated glomerulonephritis, and he had been vaccinated with an mRNA COVID-19 vaccine 6 days before rash onset. The patient was clinically diagnosed with herpes zoster infection and received systemic antiviral treatment after which his condition was relieved (18). According to the results of Diaz et al., although COVID-19 vaccination has a risk of inducing myocarditis and pericarditis, the incidence rate of these complications is extremely low. Among the 2,000,287 people who received at least one dose of a COVID-19 vaccine, 58.9% were women, 76.5% received more than one dose, 52.6% received the BNT162b2 vaccine, 44.1% received the mRNA-1,273 vaccine, and 3.1% received Ad26.COV2. Twenty people experienced vaccine-associated myocarditis [1.0 (95% CI, 0.61–1.54)/100,000], and 37 people experienced pericarditis [1.8 (95% CI, 1.30–2.55)/100,000] (19). Many international studies have shown that COVID-19 vaccination rarely induces immune thrombocytopenia and thrombosis, mainly cerebral sinus vein thrombosis, possibly owing to the side effects and the possibility of induced complications. In particular, pregnant women and pregestational women have particular concerns about the safety of vaccination against the novel coronavirus. Therefore, we should assess the safety of COVID-19 vaccines and enhance the public’s awareness about them and their confidence in them. Universal vaccination should be promoted so that we can achieve high levels of immunization in society.

Pregnant women constitute a particular population, and the D-dimer level during pregnancy has attracted much research interest. A meta-analysis by Bellesini et al. found that D-dimer may be a safe and effective diagnostic tool for identifying suspected deep vein thrombosis in pregnant women (10). A retrospective study by Zeng J et al. showed that D-dimer has good predictive value for adverse pregnancy outcomes. Pregnant women with an abnormally increased D-dimer level should be alert to the occurrence of placental abruption, postpartum hemorrhage, and severe preeclampsia (20). Gutiérrez García et al. conducted a longitudinal prospective study using the latex immunoturbidimetric method of the ACL 300 TOP automatic coagulation analyzer to measure the plasma D-dimer levels of 102 healthy pregnant women in the first, second, and third trimesters of pregnancy. The results showed that the D-dimer levels increased progressively and significantly throughout pregnancy and peaked in the third trimester. The D-dimer levels of most pregnant women in the first trimester were within the normal range, and a reference range of the D-dimer level in pregnancy was established (8). Wang et al. found similar results (21) using the Mindray EXC810 automatic coagulation analyzer to detect D-dimer concentrations and statistically analyzed the levels and change trends of coagulation parameters in healthy pregnant Chinese women in different pregnancy periods. In our study, all participants were pregnant women in the first trimester to rule out the impact of gestational age on D-dimer levels. Because vaccination has been less common in pregnant women with a history of adverse pregnancy and childbirth, the present study included this population to assess the effect of COVID-19 vaccination on D-dimer levels.

The public’s concern about the increased risk of thrombotic events post vaccination may have led to a low vaccination rate, especially in pregnant women, where thrombotic events such as venous thrombosis and pulmonary embolism may endanger the life of the mother and fetus. Crawford et al. conducted a literature search in 13 databases and found that the D-dimer level predicted pulmonary embolism with a sensitivity of 80–100% and a specificity of 23–63% (22), indicating that a low D-dimer level has some value in excluding a diagnosis of pulmonary embolism. Halaby et al. found that an increased D-dimer level was closely related to an increased risk of venous thromboembolic events, recurrent venous thromboembolism, and death (9). The present study found that vaccinated healthy pregnant women had lower D-dimer levels than those who were unvaccinated. In the adverse pregnancy history group, there was no significant difference in D-dimer levels between the vaccinated and unvaccinated subgroups. These results indicate that COVID-19 vaccination does not lead to increase D-dimer levels in healthy pregnant women or in pregnant women with a history of adverse pregnancy or childbirth. In the group of healthy pregnant women, there were differences in D-dimer levels between unvaccinated women and women who were vaccinated at different times. The women who received two or three doses had lower D-dimer levels than the unvaccinated women. There were no significant differences in D-dimer levels among women who received different vaccine brands and with different vaccination times. The combination of different brands of domestic vaccines and different periods of pregnancy may not increase the risk of thrombotic disease. The domestic vaccines are relatively safe. A previous report found that 13 days after a 24-year-old pregnant woman was vaccinated with a second mRNA COVID-19 vaccine dose, she developed a transient ischemic attack, and the blood test results showed elevated D-dimer (23). This further proves that domestic COVID-19 vaccines do not increase D-dimer levels compared with foreign vaccines, and are safer in this respect (13). Therefore, domestic COVID-19 vaccines of the same or different brands can be given pre-pregnancy and during early pregnancy.

This study had several limitations. This was a single-center retrospective study and evaluated only the D-dimer level and did not evaluate differences in other coagulation parameters. In addition, the effect of COVID-19 vaccination on the D-dimer levels of pregnant women in the second and third trimesters was not studied. However, the D-dimer levels of vaccinated and unvaccinated women were systematically compared between healthy pregnant women in the first trimester and pregnant women with a history of adverse pregnancy and childbirth. The results showed that COVID-19 vaccination did not cause increased D-dimer levels, and there were no differences in D-dimer levels among women vaccinated with different vaccine brands and at different vaccination times, providing a scientific basis for the further promotion of these vaccines. At present, few studies have evaluated the safety of COVID-19 vaccines in China, and this study can provide a reference for subsequent studies. We noted that *p* value (*p* = 0.0542) in the group of pregnant women with a history of adverse pregnancy and childbirth despite is higher than the threshold of 0.05 is near to this threshold, so we will increase the sample size of this subgroup in subsequent studies to determine whether the behavior is similar to that of healthy controls. Further more, in the future, we will work with multiple study sites to expand the sample size to further evaluate the safety of COVID-19 vaccines in terms of pregnancy trimester, disease type, and coagulation parameters. Applying rigorous scientific research methods aims to improve the credibility of COVID-19 vaccines and to promote widespread vaccination, thereby building up immunity in the population.

## Conclusion

Domestic COVID-19 vaccination does not lead to increase D-dimer levels in early pregnant women in China. Domestic COVID-19 vaccines of the same or different brands can be given pre-pregnancy and during early pregnancy.

## Data availability statement

The original contributions presented in the study are included in the article, further inquiries can be directed to the corresponding authors.

## Ethics statement

The studies involving humans were approved by The Ethics Committee of Northwest Women and Children’s Hospital. The studies were conducted in accordance with the local legislation and institutional requirements. The participants provided their written informed consent to participate in this study.

## Author contributions

YX and ZW conceived the experiments. WL analyzed the data and wrote the manuscript. WL, XF, LY, PL, XG, and QJ performed the experiments and collected data. YX, ZW, and RL guided this experiment and reviewed the article. All authors contributed to the article and approved the submitted version.

## Conflict of interest

The authors declare that the research was conducted in the absence of any commercial or financial relationships that could be construed as a potential conflict of interest.

## Publisher’s note

All claims expressed in this article are solely those of the authors and do not necessarily represent those of their affiliated organizations, or those of the publisher, the editors and the reviewers. Any product that may be evaluated in this article, or claim that may be made by its manufacturer, is not guaranteed or endorsed by the publisher.
